# Content validity of the Post-Stroke Guidance and Follow-up Booklet

**DOI:** 10.1590/0034-7167-2022-0532

**Published:** 2023-08-07

**Authors:** Bruna Fonseca Vasconcelos, Douglas de Araújo Vilhena, Luísa Fonseca Vasconcelos, Paula Mariana Munno Guimarães Corrêa, Magnania Cristiane Pereira da Costa, Juliana Nunes Santos, Thaís Peixoto Gaiad Machado

**Affiliations:** I Universidade Federal dos Vales do Jequitinhonha e Mucuri. Diamantina, Minas Gerais, Brazil; II Universidade Federal de Minas Gerais. Belo Horizonte, Minas Gerais, Brazil; III Universidade Federal de São João del-Rei. Sete Lagoas, Minas Gerais, Brazil

**Keywords:** Stroke, Rehabilitation, Validation Study, Health Education, Health Promotion, Accidente Cerebrovascular, Enfermería en Rehabilitación, Estudio de Validación, Educación en Salud, Promoción de la Salud, Acidente Vascular Cerebral, Enfermagem em Reabilitação, Estudo de Validação, Educação em Saúde, Promoção da Saúde

## Abstract

**Objectives:**

to provide sources of content validity evidence for the Post-Stroke Guidance and Follow-up Booklet.

**Methods:**

a quantitative-qualitative approach, using two Delphi method and content analysis rounds. The Educational Content Validation Instrument in Health was sent to 53 independent judges. A Content Validity Index above 0.90 was considered.

**Results:**

of the 14 nurses who participated, 64.3% have experience with stroke care, 35.7% with primary care and 64.3% with educational material production. In content analysis, judges’ suggestions were stratified into four categories: material conformation, objectivity, accuracy and perception. Spelling changes were made to the images, addition of functionality scale, adequacy of technical terms and language. The Content Validity Index in the second round showed a concordance of 0.97.

**Conclusions:**

the booklet presented satisfactory content validity sources of evidence.

## RESUMO

**Objetivos:**

prover fontes de evidências de validação de conteúdo para a Caderneta de Orientação e Acompanhamento Pós-Acidente Vascular Cerebral.

**Métodos:**

abordagem quanti-qualitativa, utilizando duas rodadas do método Delphi e análise de conteúdo. O Instrumento de Validação de Conteúdo Educativo em Saúde foi enviado para 53 juízes independentes. Considerou-se um Índice de Validade de Conteúdo acima de 0,90.

**Resultados:**

dos 14 enfermeiros que participaram, 64,3% têm experiência com atendimento a Acidente Vascular Cerebral, 35,7% com atenção primária e 64,3% com produção de material educativo. Na análise de conteúdo, as sugestões dos juízes foram estratificadas em quatro categorias: conformação, objetividade, precisão e percepção do material. Foram realizadas alterações ortográficas nas imagens, adição de escala de funcionalidade, adequação de termos técnicos e linguagem. O Índice de Validade de Conteúdo na segunda rodada demonstrou concordância de 0,97.

**Conclusões:**

a caderneta apresentou fontes de evidências de validade de conteúdo satisfatórias.

## RESUMEN

**Objetivos:**

proporcionar fuentes de evidencia de validación de contenido para el Manual de Orientación y Seguimiento Post-Accidentes Cerebrovascular.

**Métodos:**

abordaje cuantitativo-cualitativo, utilizando dos rondas del método Delphi y análisis de contenido. El Instrumento de Validación de Contenido de Educación en Salud fue enviado a 53 jueces independientes. Se consideró un Índice de Validez de Contenido superior a 0,90.

**Resultados:**

de los 14 enfermeros que participaron, el 64,3% tiene experiencia en el cuidado del ictus, el 35,7% en la atención primaria y el 64,3% en la producción de material educativo. En el análisis de contenido, las sugerencias de los jueces fueron estratificadas en cuatro categorías: conformación, objetividad, precisión y percepción del material. Se realizaron cambios ortográficos a las imágenes, adición de escala de funcionalidad, adecuación de términos técnicos y lenguaje. El Índice de Validez de Contenido en la segunda ronda mostró una concordancia de 0,97.

**Conclusiones:**

la cartilla presentó fuentes de evidencia de validez de contenido satisfactorias.

## INTRODUCTION

Knowledge about stroke is limited in the general population^( 1 )^. Even among affected people, the lack of understanding and information on the causes and consequences make it difficult to perceive the real situation in which individuals and those around them find themselves^( 2 )^. The difficulty in understanding the need and complexity of the necessary care for people affected by stroke makes it difficult to adapt to the new health condition^( 3 )^.

Despite the large number of publications on stroke, few bring proposals for conduct and/or interventions in the face of the different realities encountered. The vast majority of publications do not effectively propose actions for changes to occur^( 4 )^. Another problem that reduces the effectiveness of treatment occurs in health services that follow patients up after a stroke, since they often receive them back without having access to the information that helps in case management^( 3 )^.

Educational technologies are of great value and necessary to mediate the dissemination of information and facilitate coping with the disease^( 5 )^. Educational materials have a positive impact on the health education process and are capable of informing, guiding, modifying behaviors, promoting health and preventing illnesses^( 4 , 5 , 6 )^. To increase this impact, it is essential that the materials provide interactivity and relevant approaches, have adequate language, allow for the exchange of experiences, be attractive and present reliable and quality information^( 4 , 5 , 6 )^.

The *Caderneta de Orientação e Acompanhamento Pós-Acidente Vascular Cerebral* (COAP-AVC - Post-Stroke Guidance and Follow-up Booklet), published as an e-book by *Editora Escola Cidadã* ^( 7 )^, comes as a way to promote health, ensure the effectiveness of the care provided and give autonomy to patients and caregivers. COAP-AVC aims to align the care provided, facilitate communication between health professionals at different levels of care and between professionals and caregivers^( 7 , 8 )^.

The psychometric validity process is crucial for the choice and application of an instrument^( 9 )^. Content validity is essential for developing health education materials. It consists of measuring or addressing the absence of necessary elements and the presence of unnecessary elements, in addition to assessing their representativeness for the target population on the proposed topic^( 10 , 11 )^.

For the COAP-AVC to meet the proposed objectives and for its use to bring benefits to the public, its assessment and validity from health professionals’ perspective is necessary^( 12 )^. Content validity represents the association between interest and the dimension reached, being composed of the material design phases and its judgment by expert judges. Analysis revolves around objective adequacy and feasibility^( 11 )^. The validity process consists of assessing material applicability, by experts in the field (judges), using appropriate and accurate measurement instruments^( 12 )^.

## OBJECTIVES

To provide sources of content validity evidence for the COAP-AVC.

## METHODS

### Ethical aspects

The study was approved by the Research Ethics Committee of the *Universidade Federal dos Vales do Jequitinhonha e Mucuri* (UFVJM), under the CAAE ( *Certificado de Apresentação para Apreciação Ética* - Certificate of Presentation for Ethical Consideration).

### Design

The research used a methodological development with a quantitative and qualitative approach for educational material content validity (i.e., COAP-AVC), such as a quality improvement study (SQUIRE), carried out with professional nurses from all over Brazil with a doctorate degree or who provide assistance to people who have been affected by a stroke, using the Delphi method and content analysis^( 13 )^. The study development began in 2020 and lasted two years.

### Participants

For the initial selection of judges, resume searches were carried out on the *Plataforma Lattes* of the Brazilian National Council for Scientific and Technological Development (CNPq - *Conselho Nacional de Desenvolvimento Científico e Tecnológico* ), using the “simple search” feature, with the search mode by subject (title or keyword of the production). The databases of both doctors and other researchers were searched. In order to contemplate the three groups of themes of experience of nursing professionals in educational material judgment, the keywords were researched: (1a) nurse in the care for patients with stroke; (1b) nursing in stroke care; (2) nursing in health education material production; (3) nursing specialization in primary care. The assessment of these curricula was initially carried out by reading the abstracts, with subsequent careful analysis to verify eligibility according to inclusion criteria: (a) Brazilian nationality; (b) experience of at least five years; (c) proven performance in one of the three groups of experience themes; and (d) having an article published in the areas of interest in an indexed journal.

### Instrument

The version of the booklet submitted to content validity (COAP-AVC)^( 7 )^ consisted of fifty-eight pages, with content aimed at people affected by stroke, caregivers and health professionals. This version of COAP-AVC was published in digital book format by *Editora Escola Cidadã* , under ISBN 978-65-80725-11-3. Images were prepared manually at first and then redone in the Adobe Illustrator software image editor, in order to improve quality. To facilitate readers’ understanding, all illustrations have a simple stroke and were arranged close to the text.

The cover was illustrated with an adaptation of the Brazilian Network of Nursing and Patient Safety of São Paulo (REBRAENSP - *Rede Brasileira de Enfermagem e Segurança do Paciente de São Paulo* ) symbol and received the title “ *Caderneta de Orientação e Acompanhamento Pós-AVC - Uma Contribuição para a Segurança do Paciente* ” (Post-Stroke Guidance and Follow-up Booklet - A Contribution to Patient Safety). The first pages (1^st^ to 6^th^) of COAP-AVC consisted of a back cover, Editorial Board, catalog sheet, title page, end page, list of acronyms and summary. Later (7^th^ to 9^th^), the presentation page consisted of a description of the booklet’s function and reminders for its use, a page with a general explanation of stroke and respective differences between ischemic and hemorrhagic strokes, and a caregiver definition page and some reminders directed at them. Then (10^th^ to 13^th^), spaces were arranged to record users’ personal data, residential data and reference persons.

The booklet’s content was divided into two parts: the first was designed for users (post-stroke people and caregivers), with targeted language; the second was designed for health professionals who provide assistance to these people. The part intended for users begins with guidelines for preventing falls (14^th^ to 16^th^ page), preventing pressure injuries (17^th^ to 19^th^ page) and guidelines for basic care with devices, such as nasogastric and nasoenteric tubes, gastrostomy and urinary catheter of delay (20^th^ to 24^th^ page). All guidelines were arranged in topics and illustrated as a way to facilitate understanding. From the 25^th^ to the 31^st^ page, they were composed of spaces for filling in the notes of patients affected by stroke and/or their caregivers, of medications in use with space to record the name and time at which it should be administered, list of exams or consultations, space for the telephone directory, and presentation of useful numbers, such as the Mobile Emergency Care Service (SAMU - *Serviço de Atendimento Móvel de Urgência* ), firefighters, military police, human rights and Ethics Reporting Hotline.

The second part, aimed at health professionals, begins with an explanation of COAP-AVC’s purpose and some tips for its use (page 32). Next, the Morse Scale is described and presented, for assessing fall risk, the Braden Scale, for pressure injury risk, and the Glasgow Coma Scale, for assessing the level of consciousness, in addition to space for recording the results (33^rd^ to 38^th^ page). Pages 39 and 40 have space for registering the multidisciplinary team that provides hospital care.

From page 41 onwards, content is aimed at health professionals who follow patients up after hospital discharge. On pages 42 to 52, there are spaces for recording device assessments, wound assessments, scale assessments as well as spaces for notes by the multidisciplinary team following patients up. The last pages contain the used references and title page verses.

### Procedures

The e-mails were sent by the CNPq *Platforma Lattes* in the “contact” item. In the first round form, the judges registered their contact email, which was used to carry out the second round. The email contained: invitation letter; COAP-AVC links, available in a folder on Google Drive; form containing the Informed Consent Form (ICF); the Educational Content Validation Instrument in Health (ECVIH)^( 9 )^; and four more questions added to the bottom of Google Forms: (A) Is the booklet relevant to care (by health professionals) for a person after a stroke?; (B) Does the booklet facilitate communication between health professionals?; (C) In your view, can the booklet help improve care for people affected by a stroke?; and (D) Are the assessments and information directed to health professionals relevant?

The topics contained in the ECVIH^( 9 )^ were arranged exactly as presented in the original version of the article. Each item was made available in a multiple-choice grid, for assessment on a scale of 1 to 4 (1 = not relevant or not representative; 2 = item needs major revision to be representative; 3 = item needs minor revision to be representative; and 4 = relevant or representative item). At the end of each assessed topic (objective, structure/presentation and relevance), spaces for suggestions were made available. Access to the questionnaire was only possible after acceptance of the ICF.

### Analysis of results, and statistics

To perform agreement analysis among judges, the responses were tabulated in a Microsoft Excel® spreadsheet. Data were analyzed using SPSS software (Statistical Package for the Social Sciences, IBM Inc., USA), version 20.0. Measures of central tendency were used for the descriptive analyses, and of dispersion, for continuous variables, in addition to frequency distribution, for participant characterization. For analysis of data obtained at ECVIH, the Content Validity Index (CVI) was used (CVI = sum of responses obtained/response options x number of judges), with a criterion of agreement between judges greater than or equal to 0.90.

### RESULTS

Among the 53 eligible professional nurses contacted by email through the CNPq *Plataforma Lattes* , 14 (26.4%) agreed to participate in the study. Of these, 13 were female and 1 was male, aged between 33 and 62 years, with professional experience ranging from 5 to 40 years. Moreover, 12 have a doctorate and 2 post-doctoral degrees; 9 are active in the care for patients with stroke; 5 work in primary care; 5 have specializations in the study’s areas of interest; and 9 work with educational material production ( Table 1 ). As for location, judges from nine states of Brazil participated, six from the Northeast, four from the Southeast, and four from the South.

**Table 1:** Demographic profile of participating judges, Diamantina, Minas Gerais, Brazil, 2021

Variable	n	%
Sex		
Female	13	92.9%
Male	1	7.1%
Age		
30 to 40 years	10	71.4%
Above 50 years	4	28.6%
Education		
Doctoral degree	12	85.7%
Post-doctoral degree	2	14.3%
Work
Care for stroke patients	9	64.3%
Primary care	5	35.7%
Qualifications		
Specializations	5	35.7%
Educational material production	9	64.3%
Experience time		
5 to 10 years	7	50%
10 to 20 years	4	28.6%
More than 20 years	3	21.4%
Total	14	100%

### Delphi method – First round

The booklet’s overall CVI in the first round was 0.86, below the ideal agreement criterion established in this study (i.e., 0.90) ( Table 2 ). In response to the four questions added to the questionnaire in the first round, 12 professionals (85.7%) believe that the booklet is relevant for the care for people affected by stroke; 13 (92.9%) agree that the booklet facilitates communication between health professionals; 100% think that the booklet can help improve customer service; and 11 (78.6%) believe that assessments directed at health professionals are relevant.

**Table 2:** First round of analysis of each judge’s answers (J) in the Educational Content Validation Instrument in Health, with the Content Validity Index for each of the questions and overall average, Diamantina, Minas Gerais, Brazil, 2021

Question	J1	J2	J3	J4	J5	J6	J7	J8	J9	J10	J11	J12	J13	J14	Sum	IVC
1	2	4	4	3	4	3	4	4	2	4	3	4	4	3	48	0.87
2	2	3	3	4	3	3	4	4	3	4	3	4	4	3	47	0.84
3	2	4	3	4	4	3	3	3	2	4	3	4	4	3	46	0.82
4	3	3	3	4	4	3	3	4	3	4	3	4	4	3	48	0.86
5	3	2	2	4	4	3	3	4	3	4	3	4	4	3	46	0.82
6	3	2	3	4	3	2	3	4	3	4	3	4	4	3	45	0.80
7	3	3	4	4	3	3	3	4	3	4	3	3	4	4	48	0.86
8	2	3	4	4	3	2	3	3	3	4	3	3	4	3	44	0.79
9	4	3	4	4	3	4	3	4	2	4	3	4	4	3	49	0.88
10	4	3	4	4	3	3	3	4	2	4	3	4	4	4	49	0.88
11	4	3	4	4	3	2	3	4	2	4	3	4	4	3	47	0.84
12	4	3	4	4	4	3	3	4	2	4	3	4	4	4	50	0.89
13	3	3	4	4	3	2	3	4	2	4	2	4	4	4	46	0.82
14	4	3	4	4	4	4	3	4	4	4	4	4	4	4	54	0.96
15	4	2	4	4	3	2	3	4	4	4	3	4	4	4	49	0.88
16	2	2	4	4	4	3	4	4	4	4	3	4	4	3	49	0.88
17	3	2	4	4	4	3	4	4	3	4	3	4	4	4	50	0.89
18	4	2	3	4	4	3	4	4	4	4	3	4	4	3	50	0.89
**Sum**	**56**	**50**	**65**	**71**	**63**	**51**	**59**	**70**	**51**	**72**	**54**	**70**	**72**	**61**	**865**	**0.86**

### Content analysis – First round

The suggestions left by ten judges, in the spaces provided at the end of each topic of the questionnaire, were classified into ten initial groups, which were later grouped into four final categories, according to their intentions (i.e., conformation, objectivity, accuracy and material perception) ( Chart 1 ). In conformation, judges’ observations were included regarding the booklet size, organization, format, reference and appearance, as demonstrated in the speech of judge J2: “I found the material to be long. With guidelines for users on care and for professionals. Try to make it more objective. I think it would be more interesting to put the identification data at the beginning and then start with the care part. And at the very end the part of the scales. Users may find the booklet’s organization as-is confusing”.

**Chart 1:** Codification of contents addressed in the 10 judges’ suggestions, Diamantina, Minas Gerais, Brazil, 2021

INITIAL GROUPS	FINAL GROUPS
Size Organization Format Reference	Conformation
Writing Technical terms	Objectivity
Images Scales Additions	Accuracy
Material perception	Material perception

Objectivity included the judges’ suggestions regarding the material grammatical composition, which includes writing and use of technical terms. As suggested by Judge J6, they should: “Reassess the technical terms. Increase font size. In the part of the useful telephone numbers, in addition to the names, they could insert the Logo/icon because we have many users/relatives who do not know how to read and recognize more symbols or numbers”.

In accuracy, it brings suggestions from judges related to images and scales. As suggested by judge J11: “Still, some images do not complement the information, I suggest that they be revised to facilitate users’ understanding. These confusing images are in the fall prevention section, especially in the recommendations: free runners; bathroom rugs; risk clothing; pick up utensils”. Judge J4 said: “I suggest that a scale be used to assess functionality, so that it is periodically monitored for possible assessment of loss or gain”.

In material perception, the available spaces allowed that, in addition to suggestions, some judges expressed their opinions in relation to the booklet. For judge J3: “The educational material has guidelines and follow-up in execution of care. I do not perceive it as a promoter of reflections and behavior change”.

Based on judges’ suggestions in the first round, the booklet underwent some modifications in order to achieve higher agreement rates. Spelling changes were made to the images, addition of a scale for assessing functionality, adequacy of technical terms and language, as shown in Chart 2 .

**Chart 2:** Summary of changes made and pages, Diamantina, Minas Gerais, Brazil, 2021

Summary of changes	Pages
Regarding the booklet size, since it is aimed at two different audiences, reducing the content was not possible, but two pages were added that accentuate this division with indicative messages explaining the booklet’s purpose for each audience	13 and 37
Addition of EQVE-AVE functionality assessment scale with brief presentation to professionals	43 to 46
Update of the Glasgow Coma Scale to the version published in 2018	42
Regarding the images, some were replaced by photos indicating the necessary action	15 to 29
Addition of information items for self-care and mobility	14, 15 to 18
Spelling correction and adequacy of technical terms were performed	Whole material
Reduced explanation of scales and clearer and more objective presentation	Whole material

### Delphi method – Second round

In the second round, 12 of the 14 judges answered the questionnaire, with judges J2 and J5 not participating. It was found that all ECVIH items reached CVI concordance values greater than the 0.90 criterion ( Table 3 ). The overall CVI in the second round was 0.97, which demonstrates high agreement between the judges and that the COAP-AVC presents a satisfactory source of evidence of content validity.

**Table 3:** Second round of analysis of each judge’s answers (J) in the Educational Content Validation Instrument in Health, with the Content Validity Index for each question and overall average, Diamantina, Minas Gerais, Brazil, 2021

Question	J1	J3	J4	J6	J7	J8	J9	J10	J11	J12	J13	J14	Sum	CVI
1	4	4	4	4	4	4	4	4	4	4	4	4	48	1.00
2	4	4	4	3	4	4	4	4	4	4	4	4	47	0.98
3	4	4	4	3	4	4	4	4	3	4	4	4	46	0.96
4	4	4	4	3	4	4	4	4	3	4	4	4	46	0.96
5	2	4	4	3	3	4	4	4	4	4	4	4	44	0.92
6	3	4	4	3	4	4	3	4	4	4	4	4	45	0.94
7	4	4	4	3	4	4	3	4	4	4	4	4	46	0.96
8	4	4	4	3	4	4	3	4	4	4	4	4	46	0.96
9	4	4	4	3	4	4	4	4	4	4	4	3	46	0.96
10	4	4	4	4	4	4	4	4	4	4	4	4	48	1.00
11	4	4	4	3	4	4	4	4	4	4	4	4	47	0.98
12	4	4	4	3	4	4	4	4	3	4	4	4	46	0.96
13	2	4	4	3	4	4	4	4	4	4	4	3	44	0.92
14	4	4	4	4	4	4	4	4	4	4	4	4	48	1.00
15	4	4	4	3	4	4	4	4	4	4	4	4	47	0.98
16	4	4	4	3	4	4	4	4	4	4	4	4	47	0.98
17	4	4	4	4	4	4	4	4	4	4	4	4	48	1.00
18	4	4	4	3	4	4	4	4	4	4	4	4	47	0.98
**Sum**	**67**	**72**	**72**	**58**	**71**	**72**	**69**	**72**	**69**	**72**	**72**	**70**	**836**	**0.97**

### Content analysis – Second round

A second round of content analysis was performed for COAP-AVC. In the conformation category, J8’s comment stands out: “As the booklet has useful information for lay caregivers and for professional nursing caregivers, it may be interesting to highlight in the presentation that this booklet also considers nursing professionals as caregivers and that there will be specific content for it at the end of the booklet”.

In terms of objectivity, some terms were revised to meet J9’s comment: “Language for patients requires adaptation of technical terms, I suggest revision”. The accuracy category was adequate, with J1 commenting: “I really liked the photos! It was very realistic.” Regarding material perception, J11 commented: “I found it much more organized. I believe the changes were enough. I liked the last version sent”. J4 said that COAP-AVC is “Excellent for both patients and professionals”.

Based on judges’ suggestions in the second round, the booklet underwent some modifications, with the aim of achieving higher agreement rates. The main changes made to the final version of COAP-AVC, with page indication, are shown in Chart 3 .

**Chart 3:** Summary of changes made and pages, Diamantina, Minas Gerais, Brazil, 2021

Summary of changes	Pages
Spelling and technical term corrections	All pages
Adjustments of images that demonstrate “not letting the probe collection bag touch the ground” and “never lifting the collection bag above the waist”	Page 28
Change of order: the page destined to indication of skin injury moved to before the assessment of injury characteristics	Pages 55 to 57

## DISCUSSION

The present study aimed to provide sources of content validity evidence for COAP-AVC^( 7 )^. Two rounds of the Delphi method and content analysis were carried out, with changes made in relation to material conformation, objectivity, accuracy and perception. The final version of COAP-AVC presented a CVI of 0.97, close to the maximum, which demonstrates a high level of agreement between judges and that the booklet has satisfactory content validity evidence.

For assessing this type of material, the participation of professionals who have knowledge in educational material production, health professionals with experience in the subject and involved with the target audience, is essential. Furthermore, quantitative and qualitative procedures must be used in educational material content validity^( 10 )^.

Content analysis allows a deepening of quantitative studies, as it qualifies the subject’s experiences and perceptions about the object of study^( 11 )^. This assessment allows knowledge of material quality regarding message understanding and acceptance, adequacy of presentations, forms, styles, effectiveness, identification of adjustments and modifications^( 14 )^.

Regarding the definition of the number of judges, it is important to take into account the instrument characteristics and professionals’ availability^( 12 )^. To carry out the Delphi method, a number of experts less than ten may compromise the effective consensus and relevance of the information obtained^( 15 )^. This recommended value was exceeded in the present study, with the consultation of 14 judges in the first round, and 12 in the second.

Of the participating judges, 92.9% agree that the booklet facilitates communication between health professionals. Nursing professionals must understand the importance of continuity of care and communication between the class at different levels of care, to provide quality care without risk of damage to health^( 16 , 17 )^. Communication is a means by which it is possible to understand and share messages that are capable of influencing the behavior of those involved.

Functional health literacy can be defined as the degree to which individuals are able to obtain, process and understand basic information and services necessary for making health decisions^( 18 )^. In this regard, 94% of judges believe that the language of the final version of COAP-AVC is adequate for the target audience; 96%, that the language is appropriate for the educational material; 96%, that the language is interactive, allowing the educational process’ active involvement and that the information is correct; 98%, that the information is enlightening; 96%, that the information is necessary; and 92%, who believe that the booklet has a logical sequence of ideas.

A study found, in a sample of 160 stroke survivors, that most participants had low health-related quality of life, with greater impairment in Social role (socialization with friends, sexual relations and interference of physical condition in social life) and Family role (fun with family, feeling of being a burden on family members and influence of physical condition on personal life) domains, as well as association with reduced ability to return to work (absence of work occupation), sedentary lifestyle, functional dependence, presence of caregiver, motor sequel and lack of rehabilitation after hospital discharge^( 19 )^.

All judges believe that the booklet can help improve care for post-stroke people. The person affected by STROKE and their care-givers/relatives need constant attention and care, with follow-up throughout the process of illness and adaptation to new health conditions^( 20 )^. Based on this thought, the booklet was planned and elaborated so that it would be an alternative capable of assisting in this process. COAP-AVC is an important tool in educational support for this population, by addressing aspects of care that cover different levels of complexity. Assistance should be based on quality, continuity and longevity so that these people affected by stroke achieve a satisfactory quality of life, autonomy and significant reduction of adverse effects arising from erroneous methods of care.

It is important that everyone involved in the care for post-stroke people properly read the COAP-CVA. After dehospitalization, caregivers will have to experience the attribution of care for a prolonged period of time. Caregivers must be included in the care process, so that they can develop empowerment for care (critical awareness, emancipation, social transformation and achievement of equity in health) and for having autonomy in decision-making, transforming their family, social and economic contexts^( 21 )^. Caregivers should be given attention so that they can increase their ability to face the challenges that occur in caring for the dependent person, recognize care overload factors, list strategies that can positively interfere, facilitate family caregivers’ routine and provide opportunities for their participation in the care process. Consequently, it is expected to reduce the risk of complications, readmission of post-stroke people and improve caregivers’ quality of life.

In practice, COAP-AVC implementation is a mechanism that enables the achievement of objectives present in the Patient Safety Resolution^( 22 )^ and in the Ordinance of the Health Care Network for People with Chronic Diseases (RASPDC - *Rede de Atenção à Saúde das Pessoas com Doenças Crônicas* )^( 23 )^, since it would facilitate the maintenance of communication between teams of different levels of care and would allow the recording of information regarding patients and the actions carried out with them. Among the principles are the guidelines and objectives present in the RASPDC Ordinance, the guarantee of access and articulations of existing resources for the execution of care, assessment and periodic follow-up of these individuals, establishment of strategies that help in sustaining self-care^( 23 )^, facts that corroborate the booklet’s objectives

COAP-AVC was developed with the aim of improving care, guidance and follow-up of people affected by stroke. The psychometric validity study of this type of material is an indispensable process for its suitability and feasibility of its applicability. COAP-AVC presented sources of evidence of content validity with a high rate of agreement among judges, in addition to suggestions that contributed to material enrichment and improvement. The booklet proved to be of great value in this process, as 100% of judges reported that it contributes to knowledge in the area; 98%, that it encourages learning and is suitable for the teaching-learning process; 96%, that clarifies doubts about the topic addressed with reflection on the topic; and 92%, that encourages behavior change.

### Study limitations

Study limitations are related to the investigation only of the population of professional nurses and to having been developed at the theoretical level, being important the validity with people affected by stroke and their caregivers. As a difficulty in the validity stage through the Delphi method, there is the non-compliance of 39 of the 53 eligible professionals contacted by email, the online form of collection without face-to-face contact with experts and the delay in experts’ return within the stipulated period.

### Contributions to nursing, health, or public policies

Nursing teams and other health professionals will be able to distribute COAP-AVC as a technological resource in the care for post-stroke patients, supporting the adaptation to new health conditions. COAP-AVC was prepared with illustrations and simple language, to facilitate content understanding by readers. Thorough reviews by independent expert judges provided sources of content validity for the booklet, contributing to evidence-based practice in nursing. It is expected that the proper reading of the booklet, individually or collectively, can improve assistance in situations of functional dependence, reducing the risk of complications, home accidents and new hospitalizations.

## CONCLUSIONS

COAP-AVC presented satisfactory content validity sources of evidence. The study identified that the agreement between the 12 nurse judges was very high, with a CVI of 0.97 after two Delphi method and content analysis rounds. The booklet’s validity process underwent a careful assessment regarding the objective, structure/presentation and relevance, and raised important aspects regarding material conformation, objectivity, accuracy and perception, which were used in the adequacy of the educational material’s new version. COAP-AVC provides educational guidelines for following up people affected by stroke, with adequate scientific support for the target audience, with interactive language, logical sequence of ideas and accurate, enlightening and necessary information.

## References

[1] Silva DML, Carreiro FA, Mello R (2017). Tecnologias educacionais na assistência de enfermagem em educação em saúde: revisão integrativa. Rev Enferm UFPE.

[2] Schmidt MH, Selau CM, Soares PS, Franchi EF, Piber VD, Quatrin LB (2019). Acidente vascular cerebral e diferentes limitações: uma análise interdisciplinar. Arq Ciênc Saúde UNIPAR.

[3] Nunes HJM, Queirós PJP (2017). Doente com acidente vascular cerebral: planejamento de alta, funcionalidade e qualidade de vida. Rev Bras Enferm.

[4] Maniva SJCF, Carvalho ZMF, Gomes RKG, Carvalho REFL, Ximenes LB, Freitas CHA (2018). Tecnologias educativas para educação em saúde no acidente vascular cerebral: revisão integrativa. Rev Bras Enferm.

[5] Martins I (2019). Educação em Ciências e Educação em Saúde: breves apontamentos sobre histórias, práticas e possibilidades de articulação. Ciênc Educ (Bauru).

[6] Barros EJ, Santos SS, Gomes GC, Erdmann AL (2012). Gerontotecnologia educativa voltada ao idoso estomizado à luz da complexidade. Rev Gaúcha Enferm.

[7] Vasconcelos BF, Ramos DM, Reis MLC (2020). Caderneta de Orientação e Acompanhamento Pós-Acidente Vascular Cerebral: uma contribuição para a segurança do paciente [Internet].

[8] Vasconcelos BF, Carvalho LN, Reis MLC, Campos FF, Souza DM, Costa MCP (2020). Prevalência de internações e letalidade por acidente vascular cerebral na Santa Casa de Caridade em Diamantina-MG. Rev Connect Line.

[9] Leite SS, Áfio ACE, Carvalho LV, Silva JM, Almeida PC, Pagliuca LMF (2018). Construção e validação de instrumento de validação de conteúdo educativo em saúde. Rev Bras Enferm.

[10] Medeiros RKS, Barichello E, Vitor AF, Pinto DPSR, Ferreira MA, Santos VEP (2015). Modelo de validação de conteúdo de Pasquali nas pesquisas em Enfermagem. Rev Enferm Ref.

[11] Tibúrcio MP, Melo GSM, Balduíno LSC, Freitas CCS, Costa IKF, Torres GV (2015). Content validation of an instrument to assess the knowledge about the measurement of blood pressure. Rev Pesqui Cuid Fundam.

[12] Nora CRD, Zoboli E, Vieira MM (2018). Validação por peritos: importância na tradução e adaptação de instrumentos. Rev Gaúcha Enferm.

[13] Bardin L (2011). Análise de conteúdo.

[14] Moreira MF, Nóbrega MML, Silva MIT (2003). Comunicação escrita: contribuição para a elaboração de material educativo em saúde. Rev Bras Enferm.

[15] Marques JBV, Freitas D (2018). Método DELPHI: caracterização e potencialidades na pesquisa em Educação. Pro-Posições.

[16] Alves M, Melo CL (2019). Transferência de cuidado na perspectiva de profissionais de enfermagem de um pronto-socorro. Rev Mineira Enferm.

[17] Vieira IP, Rocha KF, Benites JE, Oliveira JHM, Oliveira Pereira T, Lescano FA (2018). Funcionalidade e qualidade de vida em pacientes pós acidente vascular cerebral. J Hum Growth Dev.

[18] Passamai MPB, Sampaio HAC, Dias AMI, Cabral LA (2012). Functional health literacy: reflections and concepts on its impact on the interaction among users, professionals and the health system. Interface Comunic Saude Educ.

[19] Silva CRR, Pimenta CJL, Viana LRC, Ferreira GRS, Bezerra TA, Costa TF (2022). Qualidade de vida relacionada à saúde específica de sobreviventes de acidente vascular encefálico: fatores associados. Rev Bras Enferm.

[20] Costa TF, Gomes TM, Viana LRC, Martins KP, Costa KNFM (2016). Stroke: patient characteristics and quality of life of caregivers. Rev Bras Enferm.

[21] Magagnin AB, Heidemann ITSB (2020). Empowerment do familiar cuidador frente ao acidente vascular cerebral no ambiente hospitalar. Rev Bras Enferm.

[22] Ministério da Saúde (BR) (2013). Resolução de Diretoria Colegiada. RDC Nº 36, de 25 de julho de 2013: Institui ações para a segurança do paciente em serviços de saúde e dá outras providências [Internet].

[23] Ministério da Saúde do Brasil (2014). Portaria nº483 de 1 de Abril de 2014: Redefine a Rede de Atenção à Saúde das Pessoas com Doenças Crônicas no âmbito do Sistema Único de Saúde (SUS) e estabelece diretrizes para a organização das suas linhas de cuidado [Internet].

